# Recent Progress in Nanobiosensors for Precise Detection of Blood Glucose Level

**DOI:** 10.1155/2022/2964705

**Published:** 2022-01-17

**Authors:** Haniye Khosravi Ardakani, Mitra Gerami, Mostafa Chashmpoosh, Navid Omidifar, Ahmad Gholami

**Affiliations:** ^1^Biotechnology Research Center, University of Medical Sciences, Shiraz, Iran; ^2^Department of Pathology, School of Medicine, Shiraz University of Medical Sciences, Shiraz, Iran; ^3^Pharmaceutical Sciences Research Center, University of Medical Sciences, Shiraz, Iran

## Abstract

Diabetes mellitus (DM) follows a series of metabolic diseases categorized by high blood sugar levels. Owing to the increasing diabetes disease in the world, early diagnosis of this disease is critical. New methods such as nanotechnology have made significant progress in many areas of medical science and physiology. Nanobiosensors are very sensible and can identify single virus particles or even low concentrations of a material that can be inherently harmful. One of the main factors for developing glucose sensors in the body is the diagnosis of hypoglycemia in individuals with insulin-dependent diabetes. Therefore, this study aimed to evaluate the most up-to-date and fastest glucose detection method by nanosensors and, as a result, faster and better treatment in medical sciences. In this review, we try to explore new ways to control blood glucose levels and treat diabetes. We begin with a definition of biosensors and their classification and basis, and then we examine the latest biosensors in glucose detection and new biosensors applications, including the artificial pancreas and updating quantum graphene data.

## 1. Introduction

Diabetes mellitus (DM) is a chronic metabolic disease categorized by a prolonged rise in blood glucose levels due to a complete or partial failure of the insulin secretion process in the absorption mechanism [1]. WHO statistics also show that DM is one of the principal causes of death worldwide, which approximately 422 million people are suffering from [2]. This disease follows a series of metabolic diseases considered by high sugar levels in the blood. This disease could lead to permanent damage and irreparable physical damage, including amputation, cardiovascular, and blindness. While there is no definitive remedy for diabetes, patients could minimize its complications by closely monitoring their blood glucose levels [3]. Constant observing of blood glucose stages by physicians and even patients themselves consider the evolution of the disease and the effectiveness of treatment methods.

For Type 2 diabetic patients, fasting glucose levels must be tightly controlled in the range from 72 to 120 mg/dL, and it should be less than 153 mg/dL 1-2 h after a meal [4]. In this study, in line with recent progress on the detection of glucose and to help control blood glucose levels, we try to review new ways to detect blood glucose levels using nanostructures and treat diabetes in this article. We first review the current methods for detecting blood sugar and the importance of using nanotechnology to improve the function of biosensors. Then, we will study the mechanism of biosensors and types of nanobiosensors for this purpose. Finally, we will mention the nanobiosensors market and the future of this technology.

## 2. Current Approaches for Blood Glucose Monitoring

At present, invasive glucose detection technology is familiar, convenient, and practical, so both hospitals and home glucometers first use the blood sampling method and then analyze it to measure blood sugar *in vitro*. In hospitals, blood is taken from people on an empty stomach in the morning, and blood glucose levels are accurately measured with an automated biochemical analyzer. Although the results of this method are accurate and can be used as an essential basis for the diagnosis of diabetes, this method is unsuitable for continuous monitoring of diabetic patients due to the tedious process, long diagnosis time, and large amount of venous blood [5]. Blood glucose monitoring refers to the monitoring of blood sugar levels over a period of time, usually with an electronic home glucose meter. However, home blood glucose monitors typically use glucose oxidase biosensors, collect blood from fingertips with a disposable paper tape, and measure blood glucose concentrations through the chemical reaction stream of the tape [6]. There are many methods for noninvasive and electrochemical approach. Optical methods include near-infrared reflector spectroscopy diagnosis of blood sugar, which can generally be divided into optical methods and microwave methods, and the other methods are polar optical rotation, Raman spectroscopy, fluorescence, optical coherence tomography, and electrochemical optical techniques, including infrared reflector spectroscopy [7, 8].

New methods using nanotechnology have made significant progress in many areas of medical science and physiology. In conjunction with biological science and engineering, this technology is used to design, build, describe, and express materials and devices as the smallest functional building in the minimum nanometer size [9]. For use in medical science and physiology, these supplies and devices are considered to cooperate with structural components of the body, cells, and tissues even at the macromolecular level with high functional properties, to create a level of overlap between biological and technical systems that did not exist before [10]. The primary purpose of nanobiosensors is to acquire information at the atomic and subatomic scales and turn it into information easily analyzed and measured [11]. These nanosensors would also be well-defined as a physical or chemical sensor built using nanoscale materials and instruments of minuscule size. These sensitive sensors could detect single virus particles or even extremely low absorptions of a material that can be inherently detrimental [12].

## 3. Mechanism of Nanobiosensors

**Figure 1:**
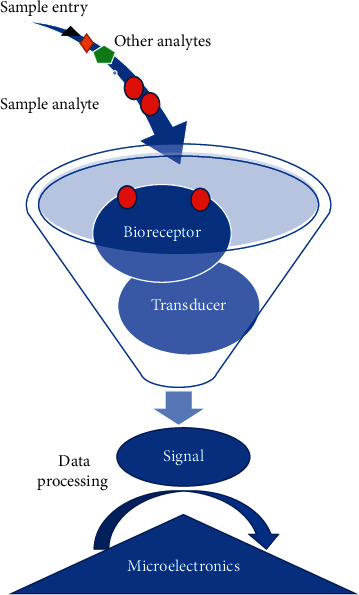
The quantitative and qualitative identification of the component by recording and presenting the signals.

Some analytical nanobiosensors can be used as real-time, simple, and actual devices to diagnose various diseases. The field of biosensors started in 1962 with the design of the glucose oxidase biosensor, which Clark and Lyons introduced [13]. Biotechnologists invented biosensors to identify bacteria and viruses and analytes based on their biomarkers or characteristics. Because of their biochemical belongings, bioreceptors are selective and sensitive in detecting biomarkers with minimal intervention with other molecules or microorganisms in the test sample, acting as sensing elements. The biosensor consists of three main components: the bioreceptor, the converter, and the signal processing system [14]. The bioreceptor portion that specifically interacts with the biomarker can be monoclonal antibodies, nucleic acids, glycans, lectins, enzymes, tissues, or whole cells. The converter section converts these interactions into a calculable signal, and then the quantitative and qualitative detection of the component is informed by displaying and recording the signals [15, 16]. As shown in Figure 1, while the sample is inserted, the desired analytes are attached to the bioreceptor. The transducer will help make a signal and the signal will be shown on screen.

Nanotechnology is involved in the expansion of biosensors through the integration of nanomaterials into biosensors. Depending on the properties of the nanomaterials, to increase the detection progressions of biosensors, it increases the external area to make more deal with analytes and improves the optical or electrical properties of converters. Conventional biosensors use more extensive size materials and can only be used for laboratory diagnosis [17]. Nanomaterials have been announced to overwhelm the restrictions of biosensors, to perform as more excellent diagnostic agents for the detection of biomarkers of an exact disease [18]. Nanomaterials increase the surface-to-volume ratio and have advanced detection abilities with high sensitivity and accuracy [19]. Biosensors-based nanomaterials are used to diagnose the disease, mainly prepared by metals, nanocomposites, carbon nanomaterials, metal oxides, and newly synthesized nanomaterial [11, 12, 20–23]. Some nanomaterials paved the way for the emergence of various classes of biosensors [24], including field effects, electrochemical sensors [25], and spectroscopy, for the quick analysis of cancer and diabetes [26]. When their limitations are reduced, nanomaterials-based biosensors become the most common method of diagnosis and can be used to quickly and sensitively diagnose diseases with new wearable, implantable, nanorobot [27], smart fabrics [28], and tattoo biosensors [29].

## 4. Glucose Monitoring

According to current methods, it is impossible to create ideal conditions for monitoring blood sugar in all situations with minimal possible complications. Therefore, nanosensors will be helpful for perfect continuous and noninvasive monitoring of blood sugar for the patient [30]. Nanosensors make continuous glucose monitoring available and can provide more accurate information about glucose detection. The most common type of glucose nanosensors was using the glucose oxidase assay for the electrical measurement of blood glucose [31]. However, nanosensors to measure glucose levels in the body require extensive research and supplementary tests, and to date, existing and studied sensors in the medical community need to improve and solve defects [31].

## 5. Accuracy of Different Nanosensors in Particle Detection

**Figure 2:**
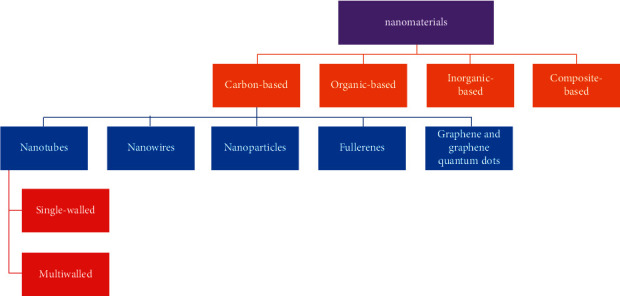
Different types of nanomaterial frequently used in nanobiosensors.

Nanosensors are measuring devices that have a detection power of at least 100 nanometers. Nanotechnologically, nanosensors are essential for (a) sensing physical and chemical variations, (b) biochemical changes in cells and observing biological molecules, and (c) determining toxins and pollutants in manufacturing and the environment, so the wide range of uses of this tool is not hidden from anyone. Nanomaterials frequently used in the structure of nanosensors include nanoscale wires (high discovery sensitivity), nanotubes of carbon (very high external area), nanoparticles, thin films, and polymer (Figure 2) [32]. The ability to measure these nanosensors to detect particles and molecules covers a wide range, for example, some examples of these nanosensors and their measurement accuracy. Magnetic nanosensors can detect definite biological molecules, for instance, proteins that possess enzymatic motion and pathogens (e.g., virus) that have low sensitivity reaction in the femtomolar range (30 ± 0.5 Fmol) [33, 34]. Metal oxide nanosensors, for example, based on gold, offer the ability to detect at least 32.5 PM, which was done by Chen et al. in a cost-effective and straightforward procedure for making nanosensors [35]. Sebastian et al. [36] revealed that the Ag sensor shows a reasonable limit for detecting 6–10 × 1.2 mm of mercury (II) ions. The mixture of Ag nanoparticles with different matrices, including fibers, silicate lattice, polymers, graphene, metal oxides, and dendrimers, provided high sensing efficiency and increased stability due to the increased application of materials. Kariuki and colleagues have manufactured an electrochemical sensor based on metal oxides in a polymeric polymer matrix. The nanoparticle-based sensor displayed a detection boundary of 1.68 mm with a widespread linear of about 10–600 mm and a great sensitivity around 7.88 mA, which has low interference in nitroaromatic-like organizational compounds [37].

Graphene, a unique two-dimensional nanostructure, can permit fast electron transfer, which has potential submissions in biosensors and electrochemical sensors [38]. They are more than graphite and double-fold carbon nanotubes. In addition, it is a conductor-like structure with zero band gaps and has two poles that cover the effect of an electric content with great charge carrier flexibility (15,000–20,000 cm^2^/Vs) [39, 40]. Porous carbon (charcoal), with its large external area and availability with a short route to transfer electron and mass, has also attracted significant attention and formed the foundation of electrochemical sensors [41]. Due to the International Union of Pure and Applied Chemistry (IUPA), spongy materials are separated into three classes based on their pore size: microporous < 2 nm, 2 nm < mesoporous < 50 nm, and macroporous > 50 nanometer. This division can be used for diagnosis and measurement [32, 42].

## 6. Types of Nanosensors for Glucose Monitoring

**Figure 3:**
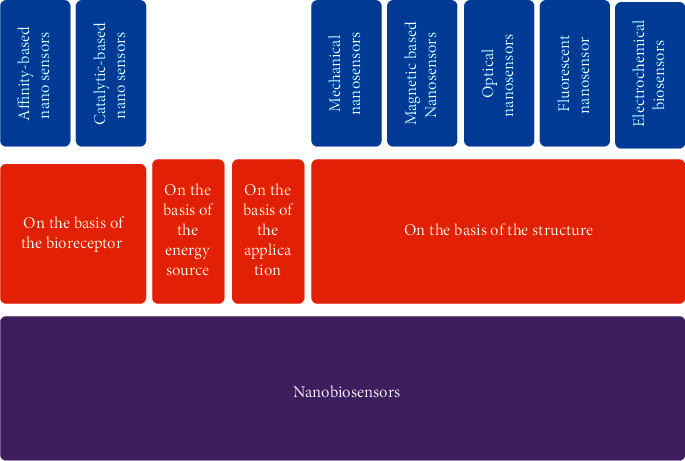
Classification of nanosensors.

There are several types and classifications of nanosensors, comparing the characteristics and main differences among the various categories and different nanomaterials used. As illustrated in Figure 3, nanosensors can be classified owing to their bioreceptor, energy source, applications, and structure [43].

### 6.1. Based on the Receptor Molecules or Bioreceptor

This classification is defined based on the type of biomolecule, which considered the main component of a nanosensor, and attaches to and detects the analyte. This category can divide the nanosensors into affinity-based nanosensors, including hormone, antibodies receptors, catalytic-based nanosensors enzyme, and microbiological cell-based sensors.

### 6.2. Based on the Energy Source

In such case, these nanosensors can be classified as active ones, which need a kind of energy source, and passive nanosensors and thermistor, which is not any energy source required, including a piezoelectric thermocouple and sensor [44].

### 6.3. Based on the Application

There are four kinds of sensors that are classified based on application: deployable nanosensors, chemical sensors, biosensors, and electrometers [45].

### 6.4. Based on the Structure

Five sorts of sensors are identified based on the structure, such as electromagnetic nanosensors, fluorescent nanosensors, optical nanosensors, magnetic nanosensors, and mechanical and/or vibrational nanosensors [44]. This category is more important than other categories in terms of construction and mechanism, and we will continue to deal with this category in more detail.

## 7. Classification of Nanosensors Based on the Structure

One of the most important basis for classification of nanosensors is based on their structure. The structural features of each nanosensor determines its capabilities for detection of blood sugar.  Table 1 lists some of the nanomaterials used in the new generation of biosensors to precisely detect blood glucose. In the following, we will review the types of nanosensors studied on this basis and their characteristics.

### 7.1. Mechanical Nanosensors

Mechanical nanosensors have qualified advantages over electromagnetic nanosensors and optical nanosensors to identify nanoscale mechanical possessions. Several kinds of mechanical nanosensors exist, like carbon nanotube- (CNT-) based nanosensors [57]. The set of this diagnostic tools is a highly positive change in the detection of glucose enzymatic electrodes, to the extent that due to the high ability to transfer electrons in CNTs and their large surface area, it has received much attention. High porous nanofiber that glucose oxidase is stabilized and immobilized on could replace the electrodes [58]. The results show that these new two-dimensional nanostructures have a much higher electron level for detection than metal electrodes, and therefore they could stabilize more enzymes and produce superior signals. Other approaches in this area include electrochemical-mediated modification of nanotubes, such as ferrocene, extensively used to transfer electrons among enzymes and the electrode [46]. These newly modified nanostructured electrodes provide improvements and surface enhancements for detection compared to their original state, called macrostructural electrodes. Biological durability is another essential factor to consider. Ten years ago, a study showed that short, single-walled CNTs are sensitive to degradation by early human neutrophils [47]. In a recent study, Saniye Soylemez et al. detect glucose using chemical sensors in carbon nanotubes based on poly4-vinyl pyridine (P4VP) and single-walled carbon nanotube compounds. To provide a glucose sensor, a glass substrate containing gold electrodes was purified with (3-Bromopropyl)trichlorosilane to achieve a covalent bond between the polymer-SWCNT compound and the glass substrate. These sensors show a large selection for glucose and immediate response (within 3 seconds) of themselves [59]. Carbon nanotubes are not the usual materials for these submissions and must be changed for use. The authors of this study focused on glucose oxidase- (GOD-) based sensors, which have been extensively considered before to test the innovative operating system. Besides, the long-lasting stability of the proposed sensor under favorable conditions was examined. One of the advantages of the glucose chemical sensor with this method is its fast response (∼3 seconds), creating a nonstop sensor. From the authors' point of view, this structure increases long-term stability and immobility and provides a great link between the active electrical layer and the biomolecule, thus developing the biocompatibility of enzyme molecules. The planned system is an enzyme-based chemical sensor system in carbon nanotubes, enabling rapid response to real-time linear glucose measurement processes. Creating an affordable, fast, and powerful glucose sensor is prospective to provide a practical application to the community [59]. In another work done by Wei Zheng et al., it is a molecularly imprinted electrochemical sensor (MIECS) based on a glassy carbon electrode (GCE) modified with carbon (CD) and chitosan (CS) points to determine glucose. In this research, trial conditions, for instance, the ratio of passive monomer molar to the model used, CD to CS volume ratio, incubation period, and wash time, are optimized. Using glucose as a model analysis, MIECS has two measurement ranges, and a relatively low diagnostic limit has been confirmed in experimental conditions. MIECS also has an excellent selection, good repeatability, and stability. The proposed sensor in this study, presented by these researchers, was successfully applied to a preglucose test in a human sample. This study presents a new selective sensor and sensitivity by connecting MIT to eco-friendly CD for rapid glucose determination. This excellent sensitivity can be due to the inactivity of the CD, which improves the practical power level and electron transfer. Combined with molecular printing technology, these sensors have tremendous power. The proposed MIECS offers the benefits of good reproducibility, affordable price, and excellent selectivity for glucose, even in the presence of common irritants, and finally, the successful MIECS pilot program leads to the detection of glucose in human serum. It may even help to innovate in normal glucose observing in real samples [60]. In another research conducted by Yingnan Qin et al. in recent years, developing enzyme-free sensors with high selectivity and sensitivity for H_2_O_2_ and glucose is a biological and applied practical scientific task. In particular, it can be used without metal nanomaterials with a big surface area and good conductivity as extremely active and selective catalysts used for molecule discovery in the enzyme. In these sensors, work has been done using the hollow frame of the machine carbon nanotube hybrids. Hollow Co_3_O_4_/NCNTs hybrids display electrochemical function when prepared to reduce H_2_O_2_ (hydrogen peroxide) in neutral solutions and oxidize glucose in alkaline solutions. These excellent electrochemical functions provide hollow Co_3_O_4_/NCNTs as a promising another to enzymes in biological usage. In summary, valuable researchers have produced Co_3_O_4_/NCNTs in H_2_/AR atmosphere only by producing precursor pyrolysis in this study. In the first stage. Co_3_O_4_ was able to provide more energetic areas and develop motion. Second, conductive carbon nanotubes can mask the low conductivity of Co_3_O_4_ nanoparticles, which can facilitate the transmission of electrons and objective molecules and inhibit the accumulation of Co_3_O_4_ nanoparticles. According to the results of this study, the modified electrode has excellent electrochemical performance with high sensitivity to H_2_O_2_ detection. Although there are still many challenges to using an enzyme-free electrochemical sensor, Co_3_O_4_/NCNTs are a promising option [48].

### 7.2. Magnetic Based Nanosensors

Magnetic nanoparticles are commonly used iron oxide to enhance sensors of glucose. These elements can be combined with other CNTs [50] or used alone [61, 62]. This method is simplified due to the magnetic sensor structure, consisting of nanoparticles enhanced with GOD on the electrode [36] and conductive nanowires on the electrode surface [63]. In both samples, the particles are adsorbed to the external electrode using magnetic fields, which is one of the advantages of using magnetically enhanced nanoparticles to construct electrode assemblages.

In one of the most recent papers, Zainovia Lockman et al. developed an amperometry biosensor created by the physical uptake of the enzyme GOD on CA-IONPs (citric acid-iron oxide nanoparticles modified), and their immobility on the screen-printed carbon electrode (SPCE) was successfully evaluated. Immobilized GOD shows excellent catalytic properties relative to glucose. IONPs were synthesized by the accelerated method and with practical CA [51].

In another research, Hong Jae Cheon et al. attached GOD to a cocktail of copper nanoparticles containing Fe_3_O_4_ magnetic nanoparticles mimics peroxidase for glucose detection. The desired glucose is efficaciously settled with the best stability, magnetic, and selectivity reusability in this strategy. This kind of biosensor based on hybrid nanoflowers likewise has a high grade of accuracy and reproducibility when used on human blood samples [64].

A study by Qing Wu et al. described a new strategy for making nuclear shell magnetic microgels by free radical polymerization induced by the cascade response of GOD and HRP. Gentle polymerization around the magnetic nanoparticle bonding line allows the soft coating of the microgel layer with amazing belongings to be used for various clinical applications. Final microgels can be conclusively used as biosensitive sensors to detect glucose colorimeters [65].

### 7.3. Optical Nanosensors

Based on single-walled carbon nanotubes, a set of optical nanobiosensors have been planned to precisely notice blood glucose stages [66]. Glucose contact lenses that use photonic crystal sensors have been advanced to monitor glucose noninvasively. This substance modulates the release rate as a result of the adsorption of exact biomolecules. The signal, produced by optical sensors, counts on the equally quantum act and tissue absorption to measure light. This nanosensor works on two distinct mechanisms. One is to turn off the fluorescence transmission signal, and the other is to charge the transfer, which is covered with GOD (the enzyme which collapses glucose molecules). Free cyanide, an electron-deficient molecule, is then sprayed on the surface of the nanotube. The electrons transfer from nanotubes by free cyanide and glow off when stimulated by infrared light. In the existence of glucose, it interacts with oxidase to produce hydrogen peroxide, which is yielded by ferric cyanide reactions to satisfy the molecule's desire for electrons. If the glucose level rises, the nanotube will detect more infrared light [44].

According to an article published by Mengyu Deng et al., core/shell nanomaterials were set as luminescence nanosensors to monitor glucose stages and intracellular oxygen closely. In this design, nanosensors are obtained by covalently embedding red oxygen-emitting and yellow-emitting reference probes in hydrophobic cores and covalently bonding water-emitting glucose probes to hydrophilic shells. This system detects glucose and oxygen simultaneously. Through different dual signals of luminescence and without the help of an enzyme, these two types of diagnostic signals did not interfere with each other. Cells stained with nanosensors, with adding glucose, a rise shows in water production. With reducing the oxygen, they offer the amount of red emission. This strategy is predictable to hint at the proposal of oxygen sensors and intracellular glucose, and it could be prolonged to many extra sensors in biological systems. In summary, the researchers developed a sensor based on polymer nanoparticles to simultaneously monitor oxygen and glucose absorptions, and these sensors possess three diverse emission colors [52].

Longway Lee et al. examined the immobility of sensory patterns on the nanoparticle external and optimized local external attentions for measuring optical glucose. A fluorescent dyeing system has been combined in various amounts in poly (2-hydroxyethyl methacrylate) hydrogels to evaluate the consequence of quencher-fluorophore concentration on the glucose response. The assay patterns on the silica nanoparticles are then immobilized by carbodiimide chemistry. Nanosensors with a varied range of dye and quenching focusses were dared for glucose response determining optimal sensor formulation. The immobilization of this system reacts to glucose on a nanosilica particle to facilitate this electron transfer during the process to obtain detectable responses even at tiny concentrations. The cumulative concentration of glucose on nanoparticles, compared to the fluorophores, leads to the highest deceptive glucose response. Nanosensors show outstanding glucose response in the physiological series and are an excellent instrument for real-time glucose tracking. Glucose-detectable nanosensors integrated into hydrogel substrates form an implantable device. These ingredients use dyes in the near-IR spectrum and can be stimulated and noticed noninvasively through tissue. The detecting group uses a distance-dependent instrument based on electrostatic connection, which is also nonmotion at the nanoparticle superficial. Glucose sensitivity in the laboratory was enhanced by changing the qualified dye concentrations close to IR and the viologen extinguishing mechanisms in this design and study, respectively. Upcoming work includes calibrating in vivo model sensors and combining added nanoparticle sensors to measure multiple analyses [67].

In another study in the same field by Vitalijs Zubkovs and his colleagues, single-walled carbon nanotubes (SWCNT) demonstrate near-infrared intrinsic fluorescence beneficial from unlimited optical stability, and tissue transparency is a reasonable basis for biosynthesis in vivo. Existing SWCNT optical sensors that depend on load transmission to transmit signals often require external interfaces that compromise the stability and biocompatibility of the sensors. This training offered a returnable near-infrared glucose sensor without intermediaries based on glucose oxidase-packed SWCNTs (GOD -SWCNTs). GOD-SWCNT increases with a sharp increase in fluorescence in the attendance of aldohexoses, with the highest answer to glucose. In previous work in this field, the physiological revealing of glucose by GOD-SWCNT was reached by adding exogenous mediators that enable optimal redox adaptation while optimizing system reversibility. This investigates bold undetected optical sensing workmanship found on local reactivity with oxygen via enzymatic pocket doping. Enzymatic pocket doping activates a response in the absenteeism of added some middle things and enables sensor reversibility. Despite these benefits, the sensor has a least response period of 3 minutes, even though a maximum of 15 minutes could achieve signal capacity. Future efforts should hence focus on refining reaction time to capture real-time glucose level fluctuations [68].

### 7.4. Fluorescent Nanosensor Based on Quantum Dots

Another well-known and convenient nanosensor is the fluorescent nanosensor founded on quantum dot graphene S and N (N-GQDs- S) (a new fluorescent nanosensor based on S and N codoped graphene quantum dots) improved by boric acid for glucose detection. S-N-GQDs are first organized by a hydrothermal procedure. Citric acid and thiourea are used as precursors in this structure, and then S, N-GQDs are developed by boric acid to make. However, this adapted system is more straightforward, cost-effective, and well-organized than the previously described expensive boric acid-based glucose sensors. A mechanism to increase fluorescence has been proposed accordingly. N-GQDs-glucose, restriction of nonradiative intramolecular motions, and fluorescent results are enhanced in these S structures, and it works based on a new conceptual PL mechanism [69]. The functional intensity of this structure increases with the limitation of molecular motion. Another example of these nanosensors is the carbon-enhanced version, and it can even be said that these nanomaterials have found their leading position as drug delivery carriers. In this respect, graphene quantum dots (GQD) are now in the spotlight. GQD is one of the newcomers to the list of carbon-based nanomaterials. The antidiabetic potentials and antibacterial of GQD are highly functional; also, they have an excellent prospect for transporting drugs through the blood-brain barrier. Drug delivery to the tumor is also imaginable with GQD [70]. Depending on the detection mechanism, GQD-based biosensors can be photoluminescence, electrochemical, and luminescence electrochemistry. Likewise, GQD fluorescence is used for bioimaging. GQDs, therefore, offer a range of biomedical applications. Their characteristics, related activities, and mechanisms are being discovered for further advances in biomedicine. It is assumed that duo-GQDs are the next-generation drug strategies for many therapeutic barriers that have not been resolved [70].

In another study, Mona Shehab and colleagues used a simple way to prepare GQD via glucose carbonation. GQDs applied with phenylboronic acid receptors have been used as a detecting material for a nonenzymatic glucose sensor. GQD spectrometry was also used as an optical sensor. GQDs are measured with a fair sensor probe due to high metering, low toxicity, water solubility, and great photochemical possessions. Another dimension of these upgraded sensors is the luminescence electrochemical glucose-sensitive biosensor based on the graphene quantum dot organized from hydrogen peroxide and graphene oxide sheets. Through this procedure, the only chemical substance which has been added was hydrogen peroxide [71]. A strong cathodic electrochemiluminescence (ECL) signal was created by cyclic voltammetry scanning on a glassy carbon electrode in GQD and potassium persulfate blend. The proposed mechanism suggested that the ECL signal was mainly reliant on reduced GQD and dissolved oxygen. In addition, it was found that the ECL signal is silenced by H_2_O_2_, the enzymatic oxidation creation of glucose. Thus, a glucose biosensor has been prepared by promoting a combination of chitosan, glucose oxidase, and GQD on a glassy carbon electrode. GQD's unique ECL specifications and GOD analysis built an ECL sensor to detect glucose sensitivity. This is especially important for studying the properties and applications of GQD [72].

### 7.5. Electrochemical Biosensors

Usually, one of the uses of electrochemical biosensors is detecting any biochemical material by converting them into electrical signals to measure them. Electrodes, biocompatible membrane supplies, supplies combined from biological element immobilization, supporting substrates, and biological elements are essential for electrochemical biosensor fabrication. The diversity and variety in biosensor fabrication circumstances combined with these ingredients and approaches have led to some electrochemical biosensors. Wang et al. planned and advanced the obvious up-to-date electrochemical biosensor [73].

Hovancova et al. defined that electrodes made of nanomaterials, particularly nanosized metals, were highly advantageous in the progress of electrochemical biosensors to sense insulin with remarkable selectivity and sensitivity. Also, Ma et al. established a gold nanoparticle-doped beta-cyclodextrin-lead metal-organic context for the sensitive purpose of insulin [74]. Nanobiosensors and electrochemical biosensors are the most appeared kinds of biosensors due to their many rewards, like time-effective process, not limited to price, high sensitivity, and lower need for sample amount [75–77]. Diverse electrochemical recognition techniques are existed (such as impedimetric, amperometry, voltammetric, and potentiometric). Fluorescence detection is one of the most uses of electrochemical glucose recognition technology [78]. One of its capabilities is the optical detection of sensors over the skin instead of implanting an electrode system. This procedure is frequently accompanied by a “smart tattoo” for the patient; the sensors are fixed in the patient's skin like regular tattoos. However, unlike conventional tattoos, these smart tattoos are temporary and must be replaced over weeks to months to account for sensor movement and signal loss due to degradation. The altered fluorescence properties of these sensors in response to blood glucose can be read using optical skin search. This method collects data continuously, so there is little or taking blood from sick people. This also decreases the risk of infection at the implant site [31, 53].

In an article by Kelvin Billingsley et al. another fluorescent nanosensor was introduced based on selected ionic optodes designed to detect small molecules. These nanosensors control the dynamic changes in the analyte concentration of the glucose model by concentrating the sensor portions in a hydrophobic core. These changes were even observed by the subcutaneous injection of nanosensors in mice. According to this research, developing these nanosensors could be an alternative and less invasive way to control glucose levels in diabetes. In addition, the development of these selectively ionic optodes optimized sensor platforms, which can detect small molecules, will pave the way for further monitoring of physiological processes [79].

In another article, Maryk Balconies et al. developed a sensor to detect small molecules by glucose-sensitive Opcodes. In this paper, a hydrophobic boronic acid was nominated to provide a rescindable fluorescence response to glucose according to the mechanism of the sensor. In this paper, they conclude that additives or different chromophores do not develop the response, though adjustments to the plastic-polymer membrane increase the device length [79].

In a more recent paper, Majid Mastery-Farahani et al. designed a new fluorescent nanosensor founded on boric acid-modified graphene doped quantum dots S and N increased the nanosensor fluorescence intensity in the attendance of glucose. Compared with other expensive sensors, this improved system is more straightforward, cost-effective, and well organized [69].

Zinc-based nanosensors are placed as a nanowire in nanotube layers and are used to detect glucose. Array and nanowire layers of ruthenium [80] and gold [81] are other materials used in nanosensors that have increased the surface and electrochemical enhancement compared to conventional electrodes. Furthermore, to produce nanoscale-modified properties at the structural electrode surface, nanosensors can be upgraded with new nanoparticles, namely, gold [49], platinum [82], and palladium [83] nanoparticles in skins to help electron transmit and surge sensor surface area.

### 7.6. Visual Nanosensor in Biofluid

Like blood, urine, tears, saliva, sweat, and interstitial fluids hold a host of ingredients (including proteins and metabolites) reflecting biological occupations and are currently increasing notice as noninvasive alternative biosources for rapid diagnostics [54]. Sweat, tears, and interstitial fluid possess a small volume with a low glucose concentration [55]. Furthermore, advanced technology is needed. To deal with the little glucose concentration in noninvasive fluids and the ultrahigh sensitivity desired for quick naked-eye detection, the nanoparticles (NPs) are a great asset to the growth of cost-effective kits. Nanoparticles (NPs), especially noble metal NPs, hold massive potential for a novel generation of extremely sensitive instrument-free colorimetric biosensors [84]. Although blood glucose monitoring is more usual, testing urine samples is helpful to confirm probable kidney dysfunctions. Glycosuria is mainly because of diabetes [85]. Naked-eye glucose detection in urine was achieved by aggregation strategy employing AuNPs functionalized with Gox [56]. Fascinatingly, the GOx bioconjugation on the AuNP also increases the enzyme constancy and resistance to degradation. When glucose surpasses 100 g/mL of concentration, the red suspension goes blue. The reaction is immediate, without any incubation time. AuNPs functionalized with the fluorophore rhodamine B isothiocyanate (RBITC) for tear glucose monitoring were investigated as fluorescent sensors. Firstly, AuNPs were synthesized with activated dextran as a stabilizing and reducing mediator and linked with aminophenyl boronic acid (ABA), forming a Schiff base. Then, the fluorophore was chemisorbed on AuNPs surface via hydrogen bonding. Glucose can create covalent bonds with boronic acid, transferring the fluorophore that, when free, can release the light signal [86].

## 8. Nanomaterials Applied in Nanosensors

The combination of biological identification fundamentals with electrochemistry raises the selection and sensitivity and shows the acceptance and success of these protein-based sensors. However, compared to nonbiological systems, there are some disadvantages to sensors based on biological cognition, such as less intrinsic stability. One of the studies in nonenzymatic glucose sensors is the direct recognition of glucose oxidation at an electrode. This method has several boundaries, including a slow response and the requirement for high application potential, which decreases its specificity. Nanomaterials play an auxiliary role in these limitations. The nanomaterials used in sensors have made great strides in this area [31]. Glucose detection was performed using copper oxide and copper nanowires [87], porous films [88], and nanostructured copper oxide/copper oxalate [89]. The direct oxidation of glucose does not need copper, but carbon in sensor structures makes the sensor perform better [90, 91]. Nanomaterial-based sensors can be considered to notice glucose due to variations in pH or charge or through a field-effect transistor (FET). As the concentration of glucose changes, the charge or pH changes near the surface.

Given the above, it seems that nanosensors based on nanotechnology will play a significant part in the analysis and treatment of diabetes. The discovery of a nanobiosensor measuring blood sugar that can accurately and instantly help physicians and patients diagnose blood sugar and provide the appropriate response is one of the severe demands of medicine and treatment globally. The discovery of precise and biocompatible nanostructures and deep integration with artificial intelligence and information technology can considerably contribute to the development of science in the future years.

## 9. The Global Market for Glucose Nanosensors

The global market for nanosensors had a total revenue of $ 432 million in 2019 and is estimated to reach $ 53.188 million by 2027. Powerful sensors can be used to detect the presence of molecules or nanoparticles of similar size. Nanosensors study data and information to understand the behavior pattern and properties of nanoparticles. The market for biosensors and nanosensors is very dynamic. Daily innovation is underway to combat market competition. The leading players in this field are the ones who dominate the market. Increasing demand from the healthcare sector has boosted nanotechnology advances by government-sponsored projects, and increased use of miniature items in many end-user industries is a crucial driver of market growth. In addition, original manufacturers in the semiconductor sector mainly use various advanced technologies in nanosensors to increase overall performance and gain a competitive advantage over other market leaders. Prominent features of nanometers, such as energy-saving, are very cost-effective and promote self-monitoring, followed by advanced investments by real nanometer manufacturers to promote advanced technologies, giving the market countless growth opportunities, and will be created in the coming years [92].

The glucose nanosensor market is expected to grow 7.5% from 2021 to 2026, reaching $ 36.7 billion by 2026. Nanomaterials, such as carbon nanotubes and indium oxide nanowires, are widely used to make biosensors or nanometer sensors based on nanotechnology. Nanobiosensors have revolutionized the future of disease diagnosis. They are essential in catalysis, optics, biomedical sciences, mechanics, magnetism, and energy sciences. For instance, wearable biosensors are the fastest-growing segment of the biosensors market. Wearable biosensors have attracted much attention because of their potential ability to change the classic medical diagnosis and ongoing health monitoring concepts. Wearable biosensor applications aim to change home care systems focused on home remedies and reduce health care costs and diagnosis time. Today, wearable biosensors bring a wave of innovation to society. Their convenience and better use can create a new level of real-time patient health exposure. This real-time data leads to better clinical decisions, better health outcomes, and more efficient use of health systems (https://www.globalbiosenssormarket.com).

## 10. Conclusion

Investigating and manufacturing nanosensors for diabetes administration is a vital part of the study and will continue in the future. To support this goal, forward work should focus on challenging real clinical specimens. These sensors had better be compared with others on the market to better establish nanosensors' advantages or weaknesses. These direct evaluations had better help justify the extra cost and struggle to overwhelm.

Industrial contests related to nanosensors are compared to standard sensors. The cost and effort of manufacturing large-scale novel sensor methods must be met with extreme precision with minimal extra cost and improved patient quality of life, due to this significant problem for overcoming strategies that combine nanomaterials into electrochemical discovery methods or sense glucose by direct oxidation. To sum up, we imagine nanotechnology to perform an essential role in glucose monitoring in the next decade. Nanotechnology formulations approved by the medical community to examine other indexes are encouraging.

## Figures and Tables

**Table 1:** Properties of nanomaterials used in nanosensors were examined to detect blood sugar.

Nanosensors	Property	References
Carbon nanotubes (CNT)	High electron transferability Extensive level It has an advanced electrical level for detection than electrodes of metal It can stabilize extra enzymes and produce superior signals Short, single-walled CNTs are sensitive to degradation by early human neutrophils	[46] [47] [48]
Graphene quantum dots	Which have increased the surface and electrochemical upgrades compared to conventional electrodes Nanosensors can be upgraded with new nanoparticles	[49]
Magnetic nanoparticles	Iron oxide has been used to upgrade sensors of glucose These elements can be collected with other structures, such as CNTs or used alone GOD has excellent catalytic properties GOD bound to copper shows mimic the activity of peroxidase to detect glucose. Therefore, the desired glucose is effectively determined with the best stability, magnetic reusability, and selectivity Magnetic microgels can be used as sensitive biosensors to detect glucose on a colorimeter	[50] [36] [51]
Nano optical sensor	They are being produced for noninvasive glucose monitoring This substance modulates the release rate in reaction to the adsorption of definite biomolecules To measure light, the optical signal depends on both quantum function and tissue absorption The greater the level of glucose, the more infrared light Working on two different mechanisms: off-fluorescence signal	[44] [52]
Electrochemical biosensors	Variety and diversity in biosensor fabrication this method collects data continuously The most attended types of biosensors	[31] [53]
Visual nanosensor	Biosources for rapid diagnostics Possessing a small volume having a low concentration of glucose Urine was achieved by aggregation strategy employing AuNPs functionalized with Gox	[54] [55] [56]

## Data Availability

The data used to support the findings of this study are included within the article.
